# Association between desloratadine and prednisolone in the treatment of children with acute symptoms of allergic rhinitis: a double-blind, randomized and controlled clinical trial^☆^

**DOI:** 10.1016/j.bjorl.2016.08.009

**Published:** 2016-09-13

**Authors:** Gustavo F. Wandalsen, Carolina Miranda, Luis Felipe Ensina, Flavio Sano, Roberto Bleul Amazonas, Joyce Macedo da Silva, Dirceu Solé

**Affiliations:** a Universidade Federal de São Paulo (Unifesp), Escola Paulista de Medicina, (EPM) Departamento de Pediatria, São Paulo, SP, Brazil; b Universidade Federal de São Paulo (Unifesp), Escola Paulista de Medicina, (EPM) Departamento de Ginecologia, São Paulo, SP, Brazil; c Fundação de Apoio à Escola Paulista de Medicina (FAP), São Paulo, SP, Brazil; d Universidade de Santo Amaro (Unisa), Clínica Médica, São Paulo, SP, Brazil; e Hospital Nipo-Brasileiro, Pediatria, São Paulo, SP, Brazil; f Universidade Estadual de Campinas (Unicamp), Campinas, SP, Brazil; g Fundação Getúlio Vargas, MBA em Marketing, Rio de Janeiro, RJ, Brazil; h Grupo NC Farma, São Paulo, SP, Brazil; i Grupo NC Farma, Pesquisa Clínica e Farmacovigilância, São Paulo, SP, Brazil

**Keywords:** Allergic rhinitis, Desloratadine, Dexchlorpheniramine, Prednisolone, Betamethasone, Rinite alérgica, Desloratadina, Dexclorfeniramina, Prednisolona, Betametasona

## Abstract

**Introduction:**

A combination of antihistamines and oral corticosteroids is often used to treat acute symptoms of allergic rhinitis.

**Objective:**

To evaluate safety and efficacy of desloratadine plus prednisolone in the treatment of acute symptoms of children (2–12 years) with allergic rhinitis, and to compare it to dexchlorpheniramine plus betamethasone.

**Methods:**

Children with moderate/severe persistent allergic rhinitis and symptomatic (nasal symptoms score [0–12] ≥ 6) were allocated in a double-blind, randomized fashion to receive dexchlorpheniramine plus betamethasone (*n* = 105; three daily doses) or desloratadine plus prednisolone (*n* = 105; single dose followed by two of placebo) for 7 days. At the beginning and end of the evaluation, the following were obtained: nasal symptoms score, extra nasal symptoms score, peak nasal inspiratory flow, blood biochemistry, and electrocardiogram. Ninety-six children of the dexchlorpheniramine plus betamethasone group and 98 of the desloratadine plus prednisolone group completed the protocol.

**Results:**

The two groups were similar regarding initial and final nasal symptoms scores, extra nasal symptoms scores and peak nasal inspiratory flow. A drop of 76.4% and 79.1% for nasal symptoms score, 86.0% and 79.2% for extra nasal symptoms score, as well as an increase of 25.2% and 24.3% for peak nasal inspiratory flow occurred for those treated with desloratadine plus prednisolone and dexchlorpheniramine plus betamethasone, respectively. There were no significant changes in blood chemistry. Sinus tachycardia was the most frequent electrocardiogram change, but with no clinical significance. Drowsiness was reported significantly more often among those of dexchlorpheniramine plus betamethasone group (17.14% × 8.57%, respectively).

**Conclusion:**

The desloratadine plus prednisolone combination was able to effectively control acute symptoms of rhinitis in children, improving symptoms and nasal function. Compared to the dexchlorpheniramine plus betamethasone combination, it showed similar clinical action, but with a lower incidence of adverse events and higher dosing convenience.

## Resumo

**Introdução:**

A associação entre anti-histamínicos e corticosteroides orais é frequentemente empregada no tratamento de sintomas agudos de Rinite Alérgica.

**Objetivo:**

Avaliar a segurança e eficácia da associação desloratadina + prednisolona no tratamento de sintomas agudos de crianças (2–12 anos) com rinite alérgica e compará-la à de dexclorfeniramina + betametasona.

**Método:**

Crianças com rinite alérgica persistente moderada/grave e sintomáticas (escore de sintomas nasais [0–12] ≥ 6) foram alocadas de modo duplo-cego e randômico para receberem dexclorfeniramina + betametasona (n = 105; três doses diárias) ou desloratadina + prednisolona (n = 105; dose única seguida por duas de placebo) por 7 dias. Ao início e final da avaliação foram obtidos: escore de sintomas nasais, escore de sintomas extra-nasais, pico de fluxo inspiratório nasal, bioquímica sanguínea e eletrocardiograma. Do total, 96 crianças do grupo dexclorfeniramina + betametasona e 98 do grupo desloratadina + prednisolona concluíram o protocolo.

**Resultados:**

Os dois grupos foram iguais com relação ao escore de sintomas nasais, escore de sintomas nasais extra-nasais e pico de fluxo inspiratório nasal iniciais e finais. Observou-se queda de 76,4% e 79,1% nos escores para escore de sintomas nasais, de 86,0% e 79,2% para escore de sintomas extra-nasais, assim como incremento de 25,2% e de 24,3% para o pico de fluxo inspiratório nasal para os grupos desloratadina + prednisolona e dexclorfeniramina + betametasona, respectivamente. Não houve alterações significativas da bioquímica sanguínea. Taquicardia sinusal foi a alteração do eletrocardiograma mais encontrada, mas sem significância clínica. Sonolência foi significantemente mais referida entre os tratados com dexclorfeniramina + betametasona do que entre os desloratadina + prednisolona (8,57% × 17,14%, respectivamente).

**Conclusão:**

A associação desloratadina + prednisolona foi capaz de controlar efetivamente os sintomas agudos de rinite em crianças, melhorando sintomas e a função nasal. Na comparação com a associação dexclorfeniramina + betametasona, demonstrou ação clínica semelhante, mas com menor incidência de eventos adversos e maior comodidade posológica.

## Introduction

ARIA initiative (Allergic Rhinitis and its Impact on Asthma) recommends that treatment of allergic rhinitis (AR) is scaled according to the severity and persistence of the disease.^1^

In general, H1 antihistamines (anti-H1) have been considered first-line drugs in the treatment of AR by exerting a significant effect on sneezing, itching and rhinorrhea, and less importantly on nasal obstruction. The second-generation H1 antihistamines have been the most recommended since they cause fewer adverse effects (sedation, anticholinergic action, among others) and have a convenient dosage (single daily dose).^1^ Among the many available in our country, the following stand out: fexofenadine, desloratadine, ebastine, levocetirizine, rupatadine and, more recently, bilastine.

Desloratadine (descarboethoxyloratadine), the primary metabolite of loratadine, is a selective antagonist of second-generation H1 receptors. It has a half-life of 27 h, its absorption is not affected by food, its metabolism and elimination are not altered by age, race and gender,^2^ and it is not affected by the simultaneous administration of macrolide antibiotics, ketoconazole and cyclosporine.^3^ In studies in patients with AR, desloratadine was effective in controlling nasal, extranasal symptoms, even after a single dose.^4^, ^5^, ^6^

Systemic corticosteroids (CS) are generally used when symptoms are not controlled with environmental or topical measures, or in more severe cases with airway compromise or major associated morbidity.^1^ Compared to topical nasal CS, the systemic administration has the advantage of reaching all parts of the nose and paranasal sinuses, even in participants with severe nasal congestion and nasal polyps.^7^

Although the simultaneous use of anti-H1 and oral CS is not recommended by ARIA in the treatment of AR, it has been widely used. An auditing of sales (units) of pharmaceuticals in 2015 showed that the association of dextrochloropheniramine and betamethasone accounts for 34.79% of sales of products available in this segment in Brazil.^8^ On the other hand, the use of anti-H1 and oral CS association in the treatment of AR is rare.

The objectives of this study were to evaluate the efficacy and safety of desloratadine + prednisolone association (oral solution; DP) in the control of acute nasal and extra-nasal symptoms in children with moderate-severe persistent AR (PAR) and to compare its action with the trade association dexchlorpheniramine + betamethasone (syrup; DB).

## Methods

This prospective, multicenter, double-blind, randomized, controlled study of parallel groups had the participation of 210 children (2–12 years) with moderate/severe PAR (1). The subjects exhibited clinical features consistent with the diagnosis of AR (recurrent nasal symptoms, and sensitization to airborne allergens by the presence of specific IgE). In addition, they had nasal symptoms scores (NSS) – ≥6 during the previous week. NSS assess nasal obstruction, rhinorrhea, sneezing and nasal itching from 0 (absent) to 3 (severe) giving a possible maximum score of 12 points. All subjects’ parents/guardians signed the informed consent document.

Patients on specific allergen immunotherapy, those with previous treatment in the last 15 days with oral corticosteroids or topical nasal or oral antihistamines, those with a chronic disease (hematopoietic, cardiovascular, renal, neurological, psychiatric and autoimmune disorders) as well as those with uncontrolled asthma, chronic rhinosinusitis and/or anatomical abnormalities of the upper airways, were not admitted to the study.

During selection (Visit 0 – V0), patients were evaluated clinically and scored according to the NSS, and the extra-nasal symptoms score – ExNSS. This evaluates itchy eyes, itchy palate, ocular hyperemia and tearing, and is scored from 0 (absent) to 3 (severe), for a maximum possible score of 12 points. Furthermore, a peripheral blood sample was collected for CBC, transaminase, urea, creatinine, bilirubin dosage, and an electrocardiogram (ECG) was performed. When subjects returned at day 5 (±2), if there were no laboratory abnormalities, patients were allocated in random order, in double-blind fashion, to the treatment groups, according to the active principle: DP or DB. At this visit, they were clinically reevaluated, and those over six years of age underwent the measurement of peak nasal inspiratory flow (PNIF) (V1). After receiving the guidelines on the daily filling of nasal symptoms and the registration of possible adverse events, patients were released and told to return in 7 (±2) days (V2) when they underwent all clinical and laboratory tests again.

### Treatment regimens

Patients were randomized by electronic CRF (Case Report Form) at the time of enrollment in the study using the criterion of blocks of six treatments, with three being DP and three DB, and received the drugs as follows: (a) DP – desloratadine (0.5 mg/mL) and prednisolone (4 mg/mL) combined in oral solution, or DB – commercially available (Celestamine^®^, Mantecorp, Brazil) a combination of dexchlorpheniramine maleate (0.4 mg/mL) and betamethasone (0.05 mg/mL) syrup. Children under 6 years who started treatment with the formulation of DP received 2.5 mL orally (vial A) complemented by two other oral doses of placebo (vials B and C), at intervals of 8 h. Patients treated with DB received three oral doses of 2.5 mL (vials A, B and C) also at 8-h intervals. Patients older than 6 years received double the dose (5 mL) employing the same distribution of vials. Vials of both treatment regimens and those of placebo were identical, and the same vehicle was used, so as to have the same flavor. The vials were labeled according to the recommendations by ANVISA.^9^ Randomization codes were broken only after analyzing the data.

### Daily nasal symptoms, self-assessment questionnaire and report of adverse events

Those responsible for the patients were instructed to fill out the diary of symptoms (sneezing, itching, runny nose and nasal obstruction) with respect to its interference in daily activities (0 = no symptoms, 1 = mild symptoms, 2 = symptoms that interfere with daily routine, but not sleep; and 3 = symptoms that interfere with sleep). The sum of scores was the score of each day of treatment.

In addition, those responsible for the patients were also asked to answer the self-assessment questionnaire regarding the use of prescribed medication (did not take, took 25%, took 50%, took 75%, took everything – 100%) on each day of treatment, as well as on the presence of any adverse events such as somnolence, headache, tremors, among others.

### Peak nasal inspiratory flow (PNIF)

Children over six years underwent PNIF at Visit 1 and at the end of treatment. PNIF measurements (In-Check^®^, Clement-Clarke, England) were conducted after patients blew their noses, in triplicate recording the highest value according to existing recommendations.^10^

### Sample calculation

Because it is a non-inferiority study of parallel groups, we employed as the primary variable the change of NSS, having admission as basis. For this purpose, we estimated a 50% reduction of NSS after treatment, and considering the maximum difference of 0.5 point between the two treatment groups, and standard deviation of 0.5 point, with an alpha error of 5%, and 95% test power would be necessary to include 86 patients per group. Estimating losses of up to 20% of patients included, the total number of patients to be included is 210; 105 in each treatment group.

### Statistical analysis

According to the nature of the variables analyzed, parametric or non-parametric tests were used, fixing the level of rejection of the null hypothesis at 5%. For the analysis to be carried out, we used SAS system (Statistical Analysis System), version 9.1.3.

The protocol was approved by the Ethics Committee of the Universidade Federal de São Paulo – Escola Paulista de Medicina and Hospital São Paulo, as well as that of all the centers involved, and it was recorded in ClinicalTrials.org PRS under number NCT 01529229. All guardians signed the informed consent, and children older than 6 years the assent form.

## Results

One-hundred and ninety-five patients completed the study (DP *n* = 98 and DB *n* = 97). Two patients from the DB group, and one of the DP group were excluded due to adverse events, three of DB and three of the DP were eliminated due to poor compliance, one of the DB group and one of the DP group were excluded due to protocol violation, one of DB and two of DP group abandoned the study, and one patient of the DB group withdrew the informed consent.

Table 1 summarizes the main clinical characteristics of the patients according to the received treatment regimen. We found that except for the use of additional drugs that were significantly higher among those in the DB group, the two groups were similar, especially regarding NSS, ExNSS, and laboratory abnormalities.

**Table 1:** Characteristics of children with moderate/severe persistent allergic rhinitis at admission, according to the treatment regimen received: DP (desloratadine + prednisolone) or DB (dexchlorpheniramine + betamethasone) – treatment intention.

Characteristic	DP (*n* = 105)	DB (*n* = 105)	Fisher *p*
*Males*	55	54	1.00

*Age (years)*			
2–6	42	32	0.19
>6	63	73	

*Concomitant medical complaints*^a^	61	54	0.41
*Concomitant medication*	84	96	*0.028*
*Baseline score of nasal symptoms*	9.0 (2.0)^b^	9.0 (2.1)^b^	0.89
*Baseline scores of extra-nasal symptoms*	5.6 (3.2)^b^	5.3 (3.1)^b^	0.51

a Associated complaints, sinusitis, asthma and headache.

b Standard deviation.

Patients treated with DP showed a reduction of 76.4% of NSS; 86.0% of ExNSS, and an increase of 25.2% of PNIF compared to baseline (Table 2). Those treated with DB showed a reduction of 79.1% of NSS; 79.2% of ExNSS, and 24.3% increase in PNIF compared to baseline (Table 2). The comparative analysis of these parameters between the two treatment regimens either at the beginning or at the end did not show significant differences.

**Table 2:** Average score (standard deviation) of nasal symptoms (NSS), of extra-nasal symptoms (ExNSS) and of peak nasal inspiratory flow (PNIF) of children with moderate/severe persistent allergic rhinitis at admission (V1) and after treatment (V2; 7 ± 2 days) with DP (desloratadine + prednisolone) or DB (dexchlorpheniramine + betamethasone).

	DP (*n* = 98)			DB (*n* = 97)		
	V1	V2	V1 − V2	V1	V2	V1 − V2
NSS	8.9 (2.0)	2.1 (2.3)	6.8	9.1 (2.1)	1.9 (2.3)	7.2
ExNSS	5.7 (3.1)	0.8 (1.2)	4.9	5.3 (3.2)	1.1 (2.2)	4.2

	DP (*n* = 61)			DB (*n* = 70)		
	V1	V2	V1 − V2	V1	V2	V1 − V2
*Peak nasal inspiratory flow (L/min)*						
PNIF	70.3 (23.9)	83.6 (26.3)	13.3	64.5 (21.9)	80.3 (25.0)	15.8

Mann–Whitney.

NSS, ExNSS and PNIF-V1: DP = DB; V2: DP = DB; V1 − V2: DP = DB.

DP and DB–NSS, ExNSS and PNIF: V1 > V2 – *p* < 0.05.

**Figure 1:**
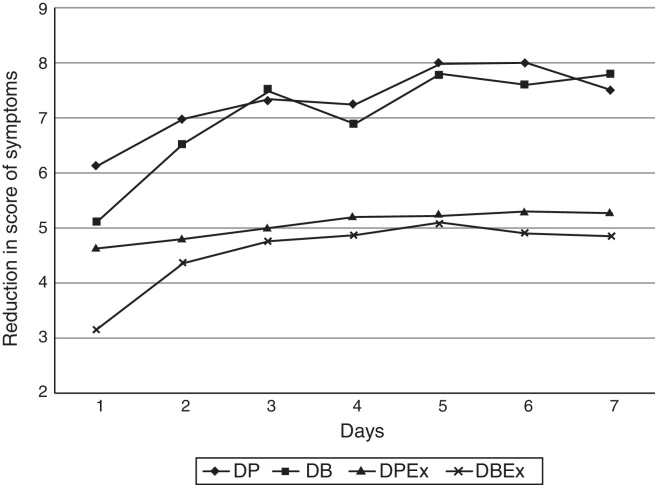
Progression of score (average) of nasal and extranasal (Ex) symptoms according to the treatment group: desloratadine + prednisolone (DP and DPEx, respectively) or dexchlorpheniramine + betamethasone (DB and DBEx, respectively) according to different days.

In Fig. 1, we observed average values of reduction in NSS and ExNSS regarding the first day of evaluation, during the 7 days of monitoring, according to the two treatment groups. We found that the two regimens provided significant reduction, but no significant differences between them.

The treatment regimen was followed by almost all the patients in both study groups. When asked about the response obtained after the treatment received, there was no significant difference between the frequency of patients who reported being much better/better after the treatment received (Table 3).

**Table 3:** Global assessment of the participant regarding received treatment: DP (desloratadine + prednisolone) or DB (dexchlorpheniramine + betamethasone).

Assessment of participant	DP	DB
	*n* (%)	*n* (%)
Much better/better	96 (98.0)	94 (96.9)
Unchanged/worse	2 (2.0)	3 (3.1)
Total	98 (100.0)	97 (100.0)

Fisher's exact test – *p* = 0.682.

Safety assessment was carried out taking the presence of significant clinical changes as basis. Only one patient of the DP group (# 40) had changes in the cardiovascular system, with no significant clinical implications. In addition, blood biochemistry revealed no patient abnormalities after treatment (data not shown). Regarding ECG findings, we found that 18 from the DB group and 29 from the DP had changes at the beginning and/or end of treatment. For all patients, these changes were not followed by significant clinical symptoms, and were considered irrelevant: mainly sinus tachycardia, physiological sinus arrhythmia, intraventricular conduction delay. Six patients in the DP group and 11 in the DB group presented changes at the end of the study, but had normal ECG tracing at the beginning, a difference that was not significant (*p* = 0.52).

As for reported adverse events, somnolence was the most reported, being significantly higher among those treated with DB (Table 4). Headache and fever had similar occurrences. However, epistaxis was reported significantly more often among DP patients (Table 4). All these events were classified as not severe (Table 4).

**Table 4:** Adverse events reported by at least 1% of patients, according to the treatment group: desloratadine + prednisolone (DP) or dexchlorpheniramine + betamethasone (DB).

Adverse event	DP	DB	*p*
	*n* (%)	*n* (%)	
Excitement	1 (0.95)	2 (1.90)	0.28
Increased appetite	2 (1.90)	5 (4.76)	0.12
Heart burn	2 (1.90)	1 (0.95)	0.28
Headache	0 (0.0)	3 (2.86)	*0.03*
Diarrhea	2 (1.90)	3 (2.86)	0.32
Pain	2 (1.90)	0 (0.0)	0.08
Abdominal pain	2 (1.90)	3 (2.86)	0.32
Epistaxis	3 (2.86)	0 (0.0)	*0.03*
Breathlessness	0 (0.0)	2 (1.90)	0.08
Fever	0 (0.0)	4 (3.81)	*0.02*
Insomnia	3 (2.86)	2 (1.90)	0.32
Irritability	1 (0.95)	2 (1.90)	0.28
Nausea	0 (0.0)	2 (1.90)	0.08
Somnolence	9 (8.57)	18 (17.14)	*0.03*
Dizziness	1 (0.95)	2 (1.90)	0.28
Cough	7 (6.60)	5 (4.76)	0.27
Vomit	3 (2.86)	2 (1.90)	0.32
ECG changes	6 (6.1)	11 (11.3)	0.52

Fisher's exact test

Italic type – significantly different.

## Discussion

Both treatment regimens were effective in controlling acute symptoms of children and adolescents with moderate/severe PAR, revealed by the reduction of NSS and ExNSS, as well as the increase of PNIF (Table 2). It is worth noting that the patients analyzed had a picture of AR of moderate to severe intensity, which often makes the treatment more challenging.^1^

Although the reduction of NSS compared to baseline values was 76.4% for those treated with DP, and 79.1% for those treated with DB, there were still patients who did not achieve full control of nasal and extra-nasal symptoms. AILA study (Allergies in Latin America) conducted to determine the prevalence of AR in the population of some Latin American countries documented that many patients identified as having AR frequently changed treatment regimens because they considered them ineffective, and the use of combination of drugs of different classes was common among them.^11^ A similar finding was observed by other researchers.^12^, ^13^

The objective measure of nasal patency was performed by measuring PNIF, an easily obtainable parameter that is reproducible and low cost. PNIF measures have proven sensitive to discriminate patients with different levels of severity of rhinitis and useful for various purposes, such as in the objective evaluation of response to treatments for allergic rhinitis.^10^ Both treatment regimens provided significant increase in nasal patency with an increase in mean values of PNIF close to 25% of baseline. This finding can be considered clinically relevant. As a comparison, nasal provocation studies consider variation in PNIF values of the order of 20% for defining relevant nasal obstruction and completion of the test.^14^, ^15^

Regarding safety evaluation of treatment regimens used, we found that the frequency of drowsiness among those treated with DB was two times higher than that on those treated with DP (Table 4). In addition, although the number of patients with ECG changes at baseline was higher among those in the DP group, this difference disappeared at the end of the study when only six patients of DP group and 11 of the DB group still had such changes.

Although first-generation, or classic, anti-H1 has been used in the treatment of allergic diseases since the 1940s, safety studies are scarce and more recent. These drugs have been developed from the same base molecule, similar to the muscarinic cholinergic antagonists, tranquilizers, antipsychotics and anti-hypertensive agents, and due to their low selectivity for H1 receptors, they interact with receptors of other active amines causing antimuscarinic, anti-α-adrenergic and anti-serotonin effects. Since they cross the blood-brain barrier easily, they bind to brain H1-receptors and interfere with neurotransmitter function of histamine causing drowsiness, sedation, fatigue, decreased readiness, worsening of cognitive function, memory and psychomotor performance.^16^ This explains the higher prevalence of drowsiness among the subjects in the DP group, since dexchlorpheniramine is representative of the classic or first generation anti-H1.^17^

It was from 1980 on that the second generation of anti-H1 emerged and started being used in large scale, with no side effects previously associated with the first-generation agents. However, the occurrence of cardiotoxic effects was associated with some of them: terfenadine and astemizole.^17^ This fact was documented to be due to competition for hepatic metabolic pathway, the cytochrome P450 system, by those drugs, ketoconazole, macrolide antibiotics, and other agents, which would result in a high circulating levels of that anti-H1 agent that were potentially cardiotoxic. This fact lead to the replacement of these anti-H1 agents by newer agents of similar chemical structure that had none of these adverse effects, but had the same power of action.^17^

Another important fact is the indiscriminate use of first generation anti-H1 agents among infants and children that came to be twice greater than drugs of the second generation. This widespread use for many years created the false impression that they were as safe as the second generation.^16^ In addition, many doctors prescribe them for their sedative effect, believing that patients will have a better sleep. This idea proved to be wrong since these first-generation anti-H1 agents prevent the patient from reaching the REM stage of sleep, making it ineffective.^18^, ^19^ The current consensus does not recommend the use of first-generation anti-H1 for the treatment of allergic rhinitis, and recommends the use of second-generation anti-H1 agents for their greater safety and lower incidence of adverse events.^19^

## Conclusion

In conclusion, although both treatment regimens have provided effective control of the symptoms of PAR, DP showed to be more advantageous due to its convenient dosage schedule (once a day) and lower frequency of adverse effects.

## Funding

Funding authority for Studies and Projects (FINEP) – Innovation and Research, Brazil, and EMS/AS – São Paulo, Brazil (Process: 01.12.0094.00; reference at FINEP-1375/10).

## Conflicts of interest

The authors declare no conflicts of interest.
